# Associations of youth mental health, parental psychological distress, and family relationships during the COVID-19 outbreak in China

**DOI:** 10.1186/s12888-022-03938-8

**Published:** 2022-04-19

**Authors:** Yashuang Bai, Xiaohan Liu, Bo Zhang, Mingqi Fu, Ning Huang, Qitu Hu, Jing Guo

**Affiliations:** 1 Department of Health Policy and Management, School of Public Health, Peking University, 100191 Beijing, China; 2 Department of Neurology and ICCTR Biostatistics and Research Design Center, Boston Children’s Hospital, Harvard Medical School, 02115 Boston, Massachusetts USA; 3 Center for Social Security Studies, Wuhan University, 430070 Wuhan, China; 4 College of Humanities Education, Inner Mongolia Medical University, 010110 Hohhot, China

**Keywords:** Parents, Youth, Mental Health, Family Relationships, COVID-19

## Abstract

**Background:**

The COVID-19 pandemic has brought significant changes in society and family life, which could be particularly difficult for parents. The present study examines the relationship between youth mental health and parental psychological distress after the first peak of the COVID-19 Outbreak in China. The parent-child and marital relationships were examined as moderators of the above relationship.

**Methods:**

Parents and their children aged 10 to 18 years were recruited for this study. The parents completed the Depression Anxiety Stress Scales-21 (DASS-21), the Strengths and Difficulties Questionnaire (SDQ), and a subset of items from the questionnaire of the COVID-19 Supporting Parents, Adolescents, and Children in Epidemics (Co-SPACE) survey of parental mental health, child’s psychological symptoms, parent-child, and marital relationship. Several multiple linear regressions were used to analyze the data.

**Results:**

The largest variance in parental mental health was explained by the child’s psychological symptoms (effect size beta = 0.27). Parent-child (effect size beta = -0.13) and marital relationship (effect size beta = -0.21) were negatively associated with parental mental health. The relationship between child’s psychological symptoms and parental mental health was moderated by marital relationship (effect size beta = -0.07). Both parent-child and marital relationships presented with a significant interaction with impact scores, while only parent-child relationships with burden scores.

**Conclusions:**

Youth mental health problems were significantly associated with parental psychological symptoms during the early phase of the COVID-19 pandemic The parent-child and marital relationship moderated the association between youth psychological symptoms and parental mental health. Interventions for alleviating parenting stress and support services that improve family relationships may be particularly effective in reducing parental psychological distress associated with future COVID-19 or related crises.

## Background

Mental health has faced a series of opposing challenges worldwide due to a fear, increased uncertainty, and social distancing since the COVID-19 outbreak [1]. One recent study showed that the incidence of mental illness has skyrocketed in China, as nearly 48.3% of Chinese citizens reached the criteria for depression and 22.6% for an anxiety disorder [2]. In particular, the risk of psychological distress may be higher among primary caregivers due to the additional needs for childcare and low levels of social support [3, 4]. Numerous studies had revealed a higher level of parenting-related stress during the pandemic lockdown than the pre-pandemic period [5, 6]. In contrast, parental stress was associated with anxiety and depressive symptoms during the COVID-19 pandemic [7–9]. For example, results from one survey showed that the prevalence of generalized anxiety disorder during the first COVID-19 lockdown phase was as high as 23.3% among parents [10]; higher than the population data indicating 14% [11]. As indicated in a multi-country study, parents reported increased COVID-related worry and emotional difficulties due to the concerns about the stability of housing situation and looking after the child at home [12]. It was also revealed that parenting children or adolescents with a mental disorder(s) might contribute to a higher risk of psychological problems among the parents [13]. One recent study recruited 6,720 families across seven European countries and showed an increase in stress, anxiety, and family conflict experienced by parents, especially if their children suffer from mental health disorders [14]. Thus, given the significant vulnerability of parents in developing mental health symptoms, this study tries to examine whether child psychological problems are associated with increased psychiatric symptoms among Chinese parents.

Studies have shown a positive link between adolescent psychological problems and parent mental health [15, 16]. From the perspective of parenting stress, it occurs when parenting demands outweigh expected parent resources [17]. Possibly, parents would experience a higher level of parenting stress when their children‘s mental health problems were identified as a source of specific difficulty and unmet need (i.e., time and financial demands) [18]. Meanwhile, a previous study has found that parenting stress was linked to higher levels of psychological distress such as depression and anxiety [19]. Besides, according to Sameroff’s transactional model [20, 21], there would be a bidirectional relationship between child behavior problems and parent depressive symptoms [22, 23]. On the one hand, parent depression is associated with escalated behavior problems among children, such as internalizing and externalizing problems. In return, increased internalizing and externalizing risks among children exacerbate parent depressive symptoms [24, 25]; however, this reverse path has received little empirical attention. The association between child mental health problems and parent psychological symptoms remains unclear. COVID-19 related evidence from China may better understand this bidirectional relationship.

Of note, family relationships may moderate the association between child mental health and parent psychological problems. According to adult attachment theory, adults with secure attachment styles are more likely to have high-quality close interactions in marriage bonds and parenthood and thus better family cohesion [26, 27]. Family cohesion is characterized by affection, warmth, connectedness, and involvement shared by family members [28]. Better family cohesion is often associated with fewer mental health problems via emotional and informational processing [28–30]. As noted by the stress-buffering model [31], support from family members may attenuate adverse effects from stressful events and reduce individuals’ negative psychological responses by personal empowerment. Furthermore, previous research has shown that a positive family relationship benefits parent mental health. In contrast, an insecure attachment with children or partners can lead to mental health issues such as stress and depression symptoms [32, 33]. Thus, it is reasonable for this study to assume that parents with better family relationships and more terrific family cohesion would report fewer mental health symptoms during the COVID-19 pandemic. However, whether the marital relationship and the parent-child relationship play similar roles in this empowerment process remains unknown.

This study aimed to examine the relationship between youth mental health and parental psychological distress in China and explore the moderating role of family relationships in the above association. The first hypothesis proposed that parents with adolescents with a high psychological distress reported more mental health symptoms. We further hypothesized that high-quality family relationships moderate the positive association between adolescent mental health and parental psychological symptoms. Finally, we assumed that these moderating effects vary between two types of family relationships, including parent-child and marital relationships.

## Methods

### Study design and participants

The current survey was conducted online between May 4th and June 7th, 2020, after the first peak of the COVID-19 outbreak, when the Chinese government has issued nationwide distancing measures, including isolation, shutting down schools, non-essential businesses, and other public places, the prohibition against holding public gatherings and events and home quarantine. We collected data through an online survey platform, “Survey Star” (https://www.wjx.cn/app/survey.aspx). To reflect parents’ psychological distress during the COVID-19 outbreaks as representatively as possible, Hubei Province, the area most affected by the pandemic, the neighboring Henan Province, and Guangdong Province southern China were selected for research investigation. Convenient sampling and cluster sampling methods were used to recruit subjects in the school. Parents with a child aged 10 to 18 years participated in this survey. In total, 746 correctly competed questionnaires were collected.

The headteachers helped us distribute the questionnaires to each student’s parent through WeChat (a web communication tool in China). Only one parent in a family was instructed to answer the questionnaire, and every WeChat account was allowed to answer the questionnaire only once to avoid repeated participation. If someone decided not to participate in the survey, they could quite easily and the web-based platform would not record it to guarantee anonymity and confidentiality. The parents were instructed to answer the questionnaires by online guidance and completed the questionnaires themselves. Participants received a small gift (e.g., 1–3 RMB) as a token of appreciation at the end of the session. All participants gave consent after being informed about the survey’s aims and joined the study voluntarily. The Ethics Committee approved the study of Peking University Medical Center.

### Questionnaires

Parental mental health was assessed with the Chinese version of Depression Anxiety Stress Scales-21 (DASS-21) [34], which was retrieved from the DASS website [35]. The DASS-21 is the short-form version of the original self-reported 42-item questionnaire. It includes three 7-item subscale dimensions of depression, anxiety, and stress symptoms. Participants responded to each question on a 4-point Likert scale from 0 (not apply to me at all) to 3 (applied to me very much or most of the time). To yield equivalent scores to the full DASS 42, the total score is determined by summing the individual items for each scale and multiplying them by 2, ranging from 0 to 126 [34]. Higher scores indicated greater severity of depression, anxiety, and stress symptoms. Cronbach’s alpha for the parent mental health scale was 0.94 in this study.

Youth mental health was assessed with the parent version of the Strengths and Difficulties Questionnaire (SDQ) and its’ extended Chinese version [36, 37]. The questionnaire has 25 positive and negative attributes over five scales, generating scores for emotional symptoms, conduct problems, hyperactivity-inattention, peer problems, and prosocial behavior. Respondents answered the 25 items with: “not true,” “somewhat true,” or “certainly true,” rated 0–2 for negatively worded items and rated inversely 2–0 for positively framed items. All scales except the prosocial score are summed to generate a total difficulties score (range 0–40), with higher scores indicating a higher level of psychological distress. In this study, the full scale of Cronbach’s alpha was 0.76.

The extended version of the SDQ also includes a brief parent-specific impact supplement [38]. The supplement questions were scored according to standard instructions to yield four impairment measures: (1) perceived difficulties, (2) chronicity, (3) impact score (social impairment plus 1 item on distress), and (4) burden score. Using a 4-point scale, responses to the perceived difficulty item were scored according to 0 = no, 1 = minor, 2 = definite, 3 = severe. Chronicity was scored as 1 = less than a month; 2 = 1 to 5 months; 3 = 6 to 12 months; 4 = over a year. The burden question was answered on a 10-point rating scale from 0 (not at all) to 10 (a great deal). For the impact score, a “0-0-1-2” rating was used so that 0 = not at all/only a little, 1 = quite a lot, 2 = a great deal. The “0-0-1-2” rating was considered to be more discriminative compared to “0-1-2-3” scoring, as the latter would require a considerable level of impairment for it to be captured [38].

The parent-child relationship and marital relationship were measured with a subset of items from the questionnaire of the Co-SPACE (COVID-19 Supporting Parents, Adolescents, and Children in Epidemics, http://cospaceoxford.org/) survey led by experts from the University of Oxford. This questionnaire was designed to track children and young people’s mental health throughout the COVID-19 crisis [39]. Seven items were included to assess the family relationships: two items about parent-child relationship, two items about marital relationship, and three items about the relationship of children with other family members. To meet the aims of this study, only the subscales of marital and parent-child relationship were used. The following questions were included: “I have an intimate relationship with my children”, “I often argue with my children”, “I have an intimate relationship with my partner”, and “My partner and I are divided about how to raise children”. The agreement to each statement was measured in four categories, representing strongly disagree (1) to agree (4) strongly. We calculated the score with inverse negative item scores. The total scores of the parent-child relationship and marital relationship were the sum of the two questions, respectively, with higher scores indicating better marital and parent-child relationships. Cronbach’s alpha of the total score of family relationships in this study was 0.60.

### Statistical analyses

Descriptive analyses were conducted to describe the parent-child relationship, marital relationship, child mental health, child difficulty impact, parent mental health, and other covariates. Means and standard deviations were used for continuous variables, while frequencies and percentages were computed for categorical variables.

Based on previous studies [13, 40], the following demographic and socioeconomic characteristics were controlled in the analyses below: province (Hubei, Henan, Guangdong), parent gender (male, female), parentage, parent education (primary and low, middle school, high school, college and above), marital status (married, divorced/other), employment (employed, farmer, unemployed), annual income (< 50000yuan, 50000-200000yuan, > 200000yuan), number of children (one, two, three or more), child gender (male, female), and child grade (grade four, grade five, grade six, grade seven, grade eight, grade nine).

The primary analyses consisted of multivariable linear regressions on parent mental health in three steps, with the same covariates used in each stage: province, parent’s gender, parentage, parent’s education, marital status, employment, annual income, number of children, child gender, and grade. Model 0 includes every predictor separately to estimate its “raw” contribution to parents’ mental health. In models 1 & 2, the predictors of exposure (i.e., parent-child relationship, marital relationship, child mental health, and child difficulty impact) were included to examine which factor would be the most potent predictor of parent mental health outcomes. In the following two linear regression analyses, the interaction variables of the standardized continuous score for the parent-child relationship, marital relationship, child mental health, and four types of child difficulty impact (perceived difficulties, chronicity, impact score, burden score) were added to the model to examine their relation with parent mental health, controlling for the same set of covariates used in the previous regressions. SPSS software version 22.0 was used to carry out all analyses.

## Results

In Table 1 are given the descriptive characteristics of the sample and the results of bivariate linear regressions of the relationship of socio-demographic characteristics and related factors with parental mental health. The mean scores of the parent-child relationship and marital relationship were 6.29 (SD = 1.29) and 6.02 (SD = 1.47), respectively. The mean score of DASS-21 was 15.86 (SD = 15.44). The average score of SDQ was 9.00 (SD = 5.14). Moreover, the mean child difficulty impact scores of the four types (perceived difficulty, chronicity, impact score and burden score) were 0.44 (SD = 0.70), 0.82 (SD = 1.39), 1.64 (SD = 3.26) and 1.12 (SD = 2.26), respectively.

**Table 1:** Descriptive characteristics of subjects and bivariate linear regression analysis for parent mental health (*N* = 746)

Variables	N (%)	Mean (SD)	B (SE)	
Province				
Hubei	309 (41.4)		-1.39 (1.15)	
Henan	222 (29.8)		-1.49 (1.24)	
Guangdong	203 (27.2)			
Parent gender				
Male	213 (28.6)			
Female	533 (71.4)		0.41 (1.25)	
Parent age		40.22 (6.12)	-0.28** (0.09)	
Parent education				
Primary school	138 (18.5)		-3.12* (1.45)	
Middle school	296 (39.7)		-0.27 (1.16)	
High school	145 (19.4)		0.65 (1.43)	
≥College	167 (22.4)			
Marital status				
Married	653 (87.5)		0.56 (1.71)	
Divorced/other	93 (12.5)			
Employment				
Employed	443 (59.4)		0.60 (1.15)	
Farmer	224 (30.0)		-2.31 (1.23)	
Unemployed	79 (10.6)			
Annual income				
Low income (< 50,000¥)	268 (35.9)		-3.23** (1.17)	
Mid income (50,000 ~ 200,000¥)	369 (49.5)		2.51* (1.13)	
High income (> 200,000¥)	109 (14.6)			
Number of children				
1	136 (18.2)		1.37 (1.46)	
2	421 (56.4)		0.94 (1.14)	
≥3	189 (25.3)			
Child gender				
Male	339 (45.4)			
Female	407 (54.6)		-1.70 (1.13)	
Child grade				
Grade four	144 (19.3)		1.31 (1.43)	
Grade five	159 (21.3)		-1.79 (1.38)	
Grade six	138 (18.5)		-2.50 (1.45)	
Grade seven	80 (10.7)		1.97 (1.83)	
Grade eight	171 (22.9)		0.66 (1.35)	
Grade nine	54 (7.2)			
Parent–child relationship		6.29 (1.29)	-3.62*** (0.41)	
Marital relationship		6.02 (1.47)	-2.94*** (0.36)	
SDQ		9.00 (5.14)	1.09*** (0.10)	
Child difficulty impact				
Perceived difficulty		0.44 (0.70)	8.62*** (0.74)	
Chronicity		0.82 (1.39)	4.24*** (0.38)	
Impact score		1.64 (3.26)	2.13*** (0.16)	
Burden score		1.12 (2.26)	2.89*** (0.23)	
DASS-21		15.86 (15.44)		

*B* coefficient, *SE* standard error; * *p* < 0.05, ** *p* < 0.01, *** *p* < 0.001

In Table 2 are presented the results of the multivariable linear regressions. In model 0, parent-child relationship, marital relationship, child mental health, and four types of child difficulty impact were significantly associated with parental mental health. In model 1, the largest variance in parental mental health was explained by the child’s mental health problems (%), with a higher level of psychological symptoms predicting more parental mental health problems (effect size beta = 0.27). Also, the parent-child (effect size beta = -0.13) and marital relationship (effect size beta = -0.21) were negatively significantly associated with parental mental health. Moreover, only two types of the child’s difficulty impact (impact score and burden score) were associated with more parent mental health symptoms, with the standardized beta ranging from 0.15 to 0.17.

**Table 2:** Multiple linear regressions with parent-child relationship, marital relationship, child mental health, and child difficulty predict parent mental health

	Model 0				Model 1			Model 2		
	B	Std.Error	Beta	B		Std.Error	Beta	B	Std.Error	Beta
Parent–child relationship	-3.59	0.43	-0.30***	-1.54		0.46	-0.13**	-1.66	0.42	-0.14***
Marital relationship	-3.42	0.39	-0.33***	-1.16		0.40	-0.21***	-2.05	0.38	-0.20***
SDQ	1.10	0.11	0.37***	0.81		0.11	0.27***			
Perceived difficulties	8.49	0.75	0.39***					1.05	1.47	0.05
Chronicity	4.17	0.39	0.38***					1.00	0.66	0.09
Impact score	3.87	0.39	0.35***					1.63	0.43	0.15***
Burden score	2.89	0.23	0.42***					1.16	0.38	0.17**

*B* coefficient, *Std. Error* standard error; *Control variables* Province, parent gender, parent age, marital status, employment, annual income, number of children, child gender, grade. All the models were adjusted for confounding; ** *p* < 0.01, *** *p* < 0.001

Table 3 reveals an interaction between the child’s mental health and marital relationship predicting parental psychological symptoms, while the interaction for parent-child relationship was not significant. The positive relationship between parental psychological symptoms and youth mental health may be buffered by marital relationship (effect size beta =-0.07). That is to say, compared to parents with a better marital relationship, parents with a worse marital relationship who reported a higher level of youth psychological symptoms would have more mental health problems. No significant interaction effects were found between youth mental health and the parent-child relationship.

**Table 3:** Multiple linear regressions for interaction effects of parent-child relationship, marital relationship, and child mental health predicting parent mental health

	Model 1			Model 2		
	B	Std. Error	Beta	B	Std. Error	Beta
Parent–child relationship	-1.93	0.59	-0.13**	-1.49	0.46	-0.13**
Marital relationship	-2.15	0.40	-0.21***	3.24	0.59	-0.21***
Child mental health	3.94	0.58	0.26***	-4.00	0.57	0.26***
Parent–child relationship* Child mental health	-0.79	0.47	-0.06			
Marital relationship* Child mental health				-1.07	0.50	-0.07*

*B* coefficient, *Std. Error* standard error; Control variables: Province, parent gender, parent age, marital status, employment, annual income, number of children, child gender, grade. All the models were adjusted for confounding;

* *p* < 0.05, ** *p* < 0.01, *** *p* < 0.001

Table 4 displays interactive effects from parent-child relationship, marital relationship, and two types of child difficulty impact (impact score and burden score) on parent psychological symptoms. Particularly, parent-child relationship (effect size beta = -0.08) and marital relationship (effect size beta = -0.08) presented significant interactions with impact score, while only parent-child relationship (effect size beta = -0.09) presented significant interaction with burden score.

**Table 4:** Multiple linear regressions for interaction effects of parent-child relationship, marital relationship, child impact score and burden score predicting parent mental health

	Model 1			Model 2			Model 3			Model 4			
	B	Std. Error	Beta	B	Std. Error	Beta	B	Std. Error	Beta	B	Std. Error	Beta	
Parent–child relationship	-2.54	0.55	-0.16***	-2.06	0.43	-0.17***	-2.28	0.55	-0.15***	-1.86	0.42	-0.16***	
Marital relationship	-2.39	0.39	-0.23***	-3.47	0.57	-0.23***	-2.12	0.38	-0.20***	-3.06	0.56	-0.20***	
Impact score	3.97	0.57	0.26***	3.95	0.60	0.26***							
Burden score							4.93	0.55	0.32***	5.05	0.57	0.33***	
Parent–child relationship* impact score	-1.18	0.51	-0.08*										
Marital relationship* impact score				-1.16	0.57	-0.08*							
Parent–child relationship* burden score							-1.28	0.48	-0.09**				
Marital relationship* burden score										- 0.85	0.50	-0.06	

*B* coefficient, *Std. Error* standard error; Control variables: Province, parent gender, parent age, marital status, employment, annual income, number of children, child gender, grade. All the models were adjusted for confounding;

* *p* < 0.05, ** *p* < 0.01, *** *p* < 0.001

## Discussion

The present study indicates that youth mental health problems and family relationships were significantly associated with parental psychological symptoms over the period of the COVID-19 outbreak in China. A marital relationship played a moderating role in youth mental health and parental psychological symptoms. Significant moderating effects of the parent-child relationship were found on the association between the impact of child difficulties and parent mental health and the association between the burden of child difficulties and parent mental health.

Findings from this study support the first hypothesis that youth mental health is associated with parental psychological symptoms, which is consistent with the results of an earlier study before the pandemic [16]. Possible reasons may be several for this observation. First, according to the family systems theory, family members are regarded as individuals who interact as part of a family system [41]. Psychopathology is theorized to arise from complex interactions among the individuals in this system, where emotional transmission and connection among family members is an ingrained feature [16, 42]. Second, the Abidin’s parenting stress model emphasizes the impact of child difficulties on the parent distress [43]. Parenting stress manifests when perceptions of parenting needs are not compensated by enough parental support [44]. When children have mental health symptoms, parents may face numerous stressors, including adjustments to their children’s internalizing and externalizing problems; a resulting incongruence between perceptions of demands and expected supports may, in turn, raise parenting stress, known to be associated with higher psychological distress [19, 45]. A recent study found that parents who had a child with a mental disorder were more likely to report higher levels of depression and anxiety [13]. The current results suggest that an intervention targeting adolescent mental health symptoms may, in turn, improve parent psychological well-being. Although it is hard to ensure the causal relationship between youth mental health and parent psychological symptoms with the cross-sectional design, the theoretical framework has provided full support for the hypothesis, which could serve as a starting point for future research to help clarify the relationship patterns using longitudinal data.

Furthermore, this study reveals significant associations among parent-child relationships, marital relationships and parent mental health. Following existing studies, the results suggest that parents with higher quality marital and parent-child relationships report fewer mental health symptoms [32, 46]. This finding is supported by the social support theory field [46], wherein the relationship perspective states that relationships enhance mental well-being by providing companionship and intimacy [47]. It has been revealed that close relationships with children and spouses may have improved parent mental health [48, 49]. As explained by the previous study, immediate parent-child relationships may provide practical and emotional support and enhance parents’ psychological well-being. In contrast, problematic relationships may evoke a sense of dissatisfaction with their parental role and be detrimental to parental well-being [50]. Similarly, a low-quality marital relationship may be a chronic stressor, and elevated stress levels could also negatively affect the mental health [51]. Thus, high-quality parent-child and marital relationships are potential resources for parents to mitigate psychological disorders during the pandemic.

Our results indicate that the marital relationship may have a significant moderating effect on the association between youth mental health and parental psychological symptoms. This interaction pattern is in the accordance with the “stress-reducing effect,” i.e., married parents, particularly in high‐quality, supportive marital relationships, are more likely to share the responsibilities of caregiving, which could reduce parenting stress for an individual parent, [51]. Thus, when rearing children with mental health symptoms, parents who have a high‐quality marital relationship and more support in caregiving from their spouse are less likely to develop parenting stress, improving their mental well-being. Finally, we find that the parent-child relationship acts as a moderator in both the association between the impact of child difficulties and parent mental health and the association between the burden of child difficulties and parent mental health. This moderating effect could be explained by “attachment anxiety” [52]. Attachment anxiety in parents comes from low quality of parent-child relationship; it was associated with common parenting satisfaction [53], which may cause elevated levels of parenting stress. Thus, the low-quality parent-child relationship could reinforce the negative effect of impact and burden of child difficulties on parent mental health symptoms. Taken together, the results suggest providing multipronged interventions. In addition to traditional psycho-education programs, it may be crucial to provide support services focused on positive marital and parent-child relationships.

### Limitations and implications

The present study had several limitations. First, all questionnaires in this study were based on parent self-reports, which may run the risk of common method biases, since no child-self reports were used. Although Harman’s single factor test suggested that common method biases were not substantial in this study, multiple methods (e.g., clinical records and interviews) and multiple informants (e.g., parents, children, and therapists) should be designed in future research to improve the validity of conclusions drawn upon the statistical results. The measurements of parent-child relationships and marital relationships were self-reported through two single items, which may lead to social desirability bias, and limit the ability to compare the findings with the previous ones. Future research should use already validated multiple-item instruments for data collection. Second, the recruitment strategy used in our study was not based on a random selection. Therefore, the generalizability of the findings to the greater significant population may be constrained. Third, adolescent mental health may be particularly associated with parent distress in the Chinese pandemic context, although such an association is likely to exist in other cultural contexts as well. Cross-cultural validations of our model should be performed in future studies. Fourth, since the survey questionnaire was released on the web communication platform by the headteachers, respondents were those who had this link access and visited the website. In this case, the eligibility of participants could not be checked. Thus, there could be a selection bias. Finally, with the cross-sectional design of the current research, it is hard to disentangle the potentially bidirectional effects of the variables. As such, longitudinal (or at least perspective) designs are highly recommended and preferable to clarify the temporal order of the relationships. Simultaneously, the cross-sectional design did not allow us to compare it to a pre-lockdown assessment. Data before the lockdown are expected in future research to examine the pandemic-related predictors on parents’ mental health.

Despite these limitations, the present study has important practical implications for prevention and intervention services promoting mental health. Evidence-based evaluative programs for parents of adolescents with mental health problems can be established on three different levels: at the level of the parent, the child, and the whole family. Firstly, considering that parents of children with mental health problems are vulnerable to psychological difficulties, special attention must be paid to these parents to help improve their mental health. For example, proactive and applicable interventions as well, as well as targeted psychoeducational programs, proposed. At the adolescent level, interventions that target adolescent internalizing and externalizing symptoms might be equally effective in reducing parenting stress. For example, either community-based or in-house programs could be designed to help treat adolescents with mental health problems.

 Additionally, given the direct and moderating effects of parent-child and marital relationships on parent mental health, practitioners could incorporate family-level components into interventions with parents. Specifically, interventions that facilitate better communication with spouses and children and teach conflict resolution skills may promote relationship satisfaction and improve psychological well-being. Finally, since the possible bidirectional relationship between adolescent mental health problems and parent psychological symptoms, it is of great importance to pay more attention to psychological screening and counseling for both adolescents and parents on a long-term and regular basis when providing mental health services.

## Conclusions

This study shows that in the Chinese population, youth mental health problems were associated with parental psychological symptoms during the early phase of the COVID-19 pandemic. The marital relationship was found to moderate the association between youth mental health and parental psychological symptoms. Moreover, the parent-child relationship played a moderating role in the association between the impact of child difficulties and parent mental health and the association between the burden of child difficulties and parent mental health. These findings suggest that it is imperative to provide positive psychological interventions that help parents alleviate their parenting stress and support services that improve marital and parent-child relationships to promote parent mental health.

## Data Availability

The datasets used and/or analyzed during the current study are available from the corresponding author on reasonable request.

## Abbreviations

COVID-19: Coronavirus Disease 2019
DASS-21: Depression Anxiety Stress Scales-21
SDQ: Strengths and Difficulties Questionnaire
SPSS: Statistical Product and Service Solutions
